# Relationship between mental health and stressors among fathers of children with chronic illnesses and cognitive structure of fathers’ stress experiences

**DOI:** 10.1038/s41598-023-48560-0

**Published:** 2023-12-18

**Authors:** Masahiro Haraguchi

**Affiliations:** Faculty of Nursing, Tokyo Healthcare University, 2-5-1 Higashigaoka, Meguro, Tokyo 152-8558 Japan

**Keywords:** Psychology, Health care

## Abstract

To clarify the relationship between stressor and mental health of the fathers with children with chronic illnesses and to examine the cognitive structure of fathers’ stress experiences. This study employs a cross-sectional research design. A self-reported questionnaire survey was conducted on 137 respondents. The dependent variables were the stressors of the fathers and depression. Focusing on the data of 51 fathers of children with chronic illness, for 21 items related to the stressful experiences had been identified in a previous study, an exploratory factor analysis using the principal factor method was performed. Logistic regression analysis results showed that the items “There are children with chronic illness” and “The large number of medical treatments required for the children” were significantly associated with the fathers’ poor mental health. In the factor analysis, the following three factors were extracted as a recognition of the fathers’ stress experiences. The fathers positively recognized their role as a father and a husband, perceiving it in a positive light despite their negative feelings. The results also suggested that it should be essential for nurses to re-evaluate those fathers who are exposed to daily stressors as care subjects, as well as to positively include them in the support activities.

## Introduction

### Background

With the recent developments in perinatal and pediatric medicine, many children with chronic illnesses who require long-term treatment are living longer. With health care reforms, the length of hospitalization has been shortened. In fact, even some patients with chronic illness who require ongoing management and those who are highly dependent on medical care can be cared for at home.

Under such medical and social circumstances, parents of children with chronic illnesses request support not only for medical care but also for other various needs, such as education and daily life activities. However, the responses to these requests are often inadequate. These parents are stressed out and suffer from psychological issues^1^. The psychological characteristics of mothers who have children with chronic illnesses have been frequently studied. It These mothers experience more difficulties raising their children as compared to mothers with healthy children^2^, and stress and coping skills affect their quality of life (QOL)^3^. Several studies have also studied the psychological characteristics of mothers who have children with chronic illnesses. The factor that influences the mothers’ levels of satisfaction with life the most is social support^4^; social support improves a mother’s ability to cope with stress, maintaining mental stability in their daily lives^5^. Mothers can also have a positive attitude toward their children’s illness and a positive outlook on life^6^. Social support has indeed an effect on coping skills and stress management as well as a direct impact on QOL. Additionally, in providing improved support for mothers, a multifaceted assessment of the personalities of mothers, patients’ conditions, and mothers’ psychological state is necessary so that they can maintain a positive attitude while their children’s chronic conditions continue. To that end, concrete support plans have been proposed. On the other hand, studies on fathers of children with chronic illnesses and the support situation among this group is unclear.

### Literature review

#### Psychological characteristics and coping behavior of fathers of children with chronic illnesses

I have reviewed papers on the psychological characteristics and coping behavior of fathers who have children with chronic illnesses published between 2000 and 2018. In addition to studies that clarified the psychological aspects, such as concerns over worsening conditions of children and their future^7–9^, studies have shown that fathers of children with chronic illnesses experienced stronger anxieties and despair as compared to fathers of healthy children when parenting and daily life become stressful^10^. These fathers were concerned about the impact of this kind of stress on their work as well^11^^,^^12^. Furthermore, it has been reported that fathers recognize themselves not as care providers but as supporters of mothers, and they feel the financial responsibility as a “bread winner”^13^. They also feel that they must stay out of the way and are unable to find a meaningful role for themselves in relation to their children^14^. Other studies have shown that, as compared to mothers, fathers tend not to show grief^15^, and they actively try to fulfill their roles as fathers and husbands even though they are experiencing difficulties and negative emotions^16–18^.

Studies on coping behavior have shown that fathers of children with chronic illnesses were not comfortable with “positive expressions,” as compared to fathers of healthy children^19^. Their coping behavior was to avoid difficult situations^20^. Contrarily, similar to mothers, these fathers have a tendency to try to get involved with their children^21^, along with a tendency to solve problems, such as acquiring information from medical books and requesting information from physicians^22^^,^^23^.

Based on these factors, fathers of children with chronic illnesses have the psychological characteristics, in which they actively try to support their children and wives as fathers and husbands even though they are shocked by their children’s health conditions and experience severe anxieties and despair. Furthermore, they have conflicting coping behavior, that is, escaping reality and yet facing reality. These results indicate that fathers need to be considered as a target of nursing based on their psychological characteristics and coping behavior^24^.

### Acknowledging stress in fathers of children with chronic illnesses

Fathers of children with chronic illnesses experience complex emotions toward their children and family, including severe anxiety and despair^25^. They often have additional concerns about their work ^11^^,^^12^ and financial responsibilities^13^. These fathers had difficulty identifying their own values^14^. In addition, they have a negative psychological characteristic in which they use self-contained problem-managing strategies against stressors and are uncomfortable expressing their emotions and asking for help from others^26^. Additionally, fathers try to fulfill their roles as fathers and husbands^27^ as they care for their wives and children^16^, try to provide support for their children “behind the scenes”^17^, and “stand up” for their children while maintaining their own identity^18^. After some period, these fathers begin living in the same manner as fathers of healthy children, or even become more active in the society^12^. There is a positive correlation between negative emotions felt by fathers and self-growth^28^. In contrast to the coping behavior of avoiding the reality of a situation, fathers also exhibit a positive coping behavior in which they actively try to solve problems^24^.

The stress experiences and characteristics of fathers of children with chronic illnesses in Japan: Under stress, fathers of children with chronic illnesses experience anxieties, despair, and difficulties, while negatively viewing the impact of the situation on work and changes in the family life. However, these fathers also have a positive view and aim to fulfill their roles as fathers and husbands, supporting their children and wives, and contributing to the society with their experiences. Furthermore, fathers often achieve self-growth through their experiences. As such, by focusing on the positive characteristics of fathers of children with chronic illnesses, not just their negative views, nursing based on a positive psychological approach can be useful^24^.

In this study, we focused on Sense of Coherence (SOC), which is one of the approaches in Positive Psychology. SOC is based on the theory of health salutogenesis, as proposed by Antonovsky, emphasizing the importance of focusing on the positive aspects rather than eliminating the negative ones, even in stressful situations and crises^29^. SOC is considered to possess the capacity to (1) flexibly and appropriately cope with stressors and (2) enable individuals to maintain their quality of life and well-being even in the presence of illness or disability^30^. Therefore, previous studies^31^^,^^32^ have elucidated how parental SOC is related to stress, the child’s health condition, and quality of life (QOL). However, there is a current scarcity of research specifically focused on fathers of children with chronic illnesses. The impact of stressors on a father’s mental health, the relationship between fathers’ mental health and stressors, and the cognitive structure of fathers’ stress experiences remain unclear. In the present study, the impact of stressors experienced on the fathers’ mental health (both fathers of healthy children and of children with chronic illness) was analyzed. Furthermore, this study aimed to clarify the cognitive structure of the stress experiences of fathers who have children with chronic illness.

## Methods

### Procedures and participants

The present study had a cross-sectional research design. We conducted a self-reported questionnaire survey on 425 fathers of children with chronic illnesses and 537 fathers of healthy children from April to August 2020. As a condition for selecting fathers of chronic illnesses children are those aged 0–16 years old who require medical treatment, who have illnesses that require long-term life restrictions and management due to contraction, and who are currently living at home. Those whose children were hospitalized at the time of the survey request were excluded. As a condition for selecting fathers of healthy children, fathers of children aged 0–16 whose children attend nursery schools, kindergartens, elementary schools, and junior high schools were selected.

Informed consent was obtained from the fathers of chronic illness children and the fathers of healthy children using the following procedure.

Requests for fathers of chronically ill children were explained verbally to the facility representative.

As for the request to the father of a healthy child, the purpose of the research was verbally explained to the representative of the facility/organization based on the survey request form over the phone.

After obtaining consent, a survey request form regarding the purpose of the research was mailed to the representative.

The representative of each organization handed over to the research participants a request for cooperation in the survey, which included a URL for accessing the input form.

We decided not to disclose to the self-help group whether the candidate for research subjects agreed to participate.

In addition, we considered that the research subjects’ responses to the questionnaire were consent to the research.

To prevent multiple participation in the Web survey, respondents were asked to enter their e-mail address after accessing the URL.

In accordance with the purpose of the study, the sample size was assumed to be medium, with a power of 0.8 and a significance level of 5%^33^. In addition, independent variables × 10 or more were calculated based on the sample size criteria^34^. The anonymous self-administered questionnaire was either sent using a closed online method or by mail.

### Measures

We prepared a questionnaire using survey questions as follows and then collected the responses.

### Fathers’ basic attributes (fathers of children with chronic illnesses and healthy children) (Table 1)

**Table 1:** Respondent’s characteristics.

Terms	Total (n = 137)			Father’s of children with chronic illnesses (n = 51)			Father’s of healthy children (n = 86)			*p*-value
	n (%)	Mean (SE)	Median (IQR)	n (%)	Mean (SE)	Median (IQR)	n (%)	Mean (SE)	Median (IQR)	
Age^a^	137 (100.0)	40.53 (± 5.98)	40 (36–44)	51 (37.2)	41.56 (± 6.32)	40 (37–44)	86 (62.7)	39.94 (± 5.76)	40 (35.25–44)	.270
Spouse										
Presence	137 (100.0)			51 (37.2)			86 (62.7)			
absence	0									
Number of family members living together^a^		3.88 (+ 1.05)	4 (3–4)		4.09 (± 1.17)	4 (3–5)		3.76 (± 0.95)	4 (3–4)	.232
Number of children^a^		1.85 (± 0.84)	2 (1–2)		2.00 (± 0.89)	2 (1–3)		1.77 (± 0.80)	2 (1–2)	.141
Average parenting time per day^b^										
Weekday										
3 h or less	111 (81.0)			37 (27.0)			74 (54.0)			.098
4 h or more	26 (19.0)			14 (10.2)			12 (8.8)			
Holiday										
3 h or less	44 (32.1)			15 (10.9)			29 (21.2)			.705
4 h or more	93 (67.9)			36 (26.3)			57 (41.6)			
Taking leave for childcare^b^										
Yes	111 (81.0)			43			68			.649
No	26 (19.0)			8			18			
Children age^a^										
First-born child	137	7.98 (± 4.90)		51	9.86 (± 4.67)		86	6.79 (± 4.97)		< .001
Children sex										
First-born child										
Male	76 (55.5)			27 (19.7)			49 (35.8)			
Female	61 (44.5)			24 (17.5)			37 (27.0)			
First-born child school attendance^b^										
Preschool child	56 (40.9)			11 (8.0)			45 (32.9)			< .001
School child	81 (59.1)			40 (29.2)			41 (29.9)			
Final educational background^b^										
Other than university / graduate school	37 (27.0)			18			19			.108
University / Graduate School	100 (73.0)			33			67			
Occupation^b^										
Professional / technical	78 (56.9)			34			44			
Non-professional / technical	59 (43.1)			17			42			
Annual personal income (yen)^a^		645.59 (± 287.77)	600 (460–800)		641.76 (± 337.69)	550 (435–800)		647.91 (± 254.94)	600 (500–800)	.457
Annual household income (yen)^a^		814.48 (± 349.80)	800 (600–1000)		729.80 (± 361.17)	600 (480–1000)		865.89 (± 334.49)	800 (700–1000)	.012

^a^Mann–Whitney U test.

^b^Fisher’s exact test.

Data on fathers’ age, family structure, occupation, financial status, and parenting time were obtained.

#### Basic attributes of children (fathers of children with chronic illnesses and healthy children)

Data on children’s age, sex, and school status were gathered.

### Attributes of childrens’ illnesses (fathers of children with chronic illnesses) (Table 2)

**Table 2:** Characteristics of children with chronic illnesses.

Terms		n (%)	Mean (SE)
Disease classification	Malignant neoplasm	2 (2.4)	
(※Multiple selection)	Kidney	1 (1.2)	
	Respiratory	15 (17.9)	
	Heart	17 (20.2)	
	Endocrine	4 (4.8)	
	Collagen	1 (1.2)	
	Blood	2 (2.4)	
	Nerves and muscles	4 (4.8)	
	Digestive	4 (4.8)	
	Chromosome / heredity	6 (7.10)	
	Skin	1 (1.2)	
	Allergy	19 (22.6)	
	Others	8 (9.5)	
Medical treatment status by birth order	First-born child	46 (68.7)	
(※Multiple selection)	Non First-born child	21 (31.3)	
Hospitalization (total number of times)			7.48 ± 10.66
	≧2 times	31 (60.8)	
	< 2 times	20 (39.2)	
Surgery (total number of times)			5.29 ± 5.24
(※ including catheterization)	≧2 times	21 (41.1)	
	< 2 times	30 (58.9)	
Commuting to hospital	Year		10.7 ± 16.58
	≧ 1 time/month	19 (37.3)	
	< 1 time/month	32 (62.7)	
Medical treatment	Oxygen	7 (6.0)	
(※Multiple selection)	Suction	9 (7.7)	
	Tube nutrition	6 (5.1)	
	Central IV nutrition	4 (3.4)	
	Blood glucose measurement	3 (2.6)	
	Insulin injection	1 (0.9)	
	Oral administration of drugs	44 (37.6)	
	External medicine	17 (14.5)	
	Inhalation	18 (15.4)	
	Others	8 (6.8)	
	≧3 medical treatment	18 (35.3)	
	< 3 medical treatment	33 (64.7)	

The name of the illness, the number of hospitalizations and operations, the frequency of outpatient visits, medical treatments, and care status were obtained.

### Mental health of fathers (fathers of children with chronic illnesses and healthy children) (Table 1)

I used the Kessler 6 scale (K6) as an index of mental health, which expressed the stress reactions of fathers. K6 is a scale that measures psychological stress reactions. It consists of six questions with the scores ranging between 0 and 24. A higher score means poorer mental health. The reliability and validity of the scale were confirmed previously^35^. This study’s Cronbach’s alpha was 0.87.

### Cognitive structure of fathers’ stress experiences (fathers of children with chronic illness)

For the 21 questionnaire items were developed through a qualitative analysis of interview data related to the stress experiences of fathers of children with chronic illnesses^24^, the respondents were asked whether they ever felt described item, and they scored each item from 1 (always felt) to 5 (not felt at all). Furthermore, regarding the questionnaire items, we have conducted extensive discussions and considerations with pediatric nursing researchers and practitioners to ensure content validity by optimizing the wording.

### An examination of the influence of stressor on father’s mental health

Based on a previous study^36^, the fathers of children with chronic diseases and those of healthy children were assigned to the high (K6 score of ≥ 5) and low (K6 score of ≤ 4) depression groups, which were used as the dependent variables. Then, logistic regression analysis was performed using the stepwise method with the variables shown below as independent variables. Independent variables were classified into the following two groups based on their correlation with each variable and basic attributes: age, number of people living together, number of children, whether or not childcare leave is taken, whether or not there is a child with a chronic disease, first child’s attendance at school, educational background, industry, individual annual income, and household annual income.

### Examination of the cognitive structure of the stress experiences of fathers with children with chronic illness

An exploratory factor analysis using the principal factor method (promax rotation) was performed. Two items with factor lodging of ≤ 0.40 were excluded. The Cronbach’s alpha coefficient was calculated to verify internal consistency.

### Ethical considerations

This study was conducted following the Ministry of Health, Labour and Welfare’s “Ethics Guidelines for Medical Research involving Human subjects”^37^ with the proper ethical considerations.

This study was conducted with the approval of the ethical review board of the Tokyo Healthcare University Committee chief investigator’s institution (Approval No.: 31-57B). This study has no conflict of interest to declare.

### Statistical analyses

The analyses were carried out with SPSS 23.0J for Windows (SPSS, Tokyo, Japan) with significance set at 5% (two-sided).

## Results

I received responses from 51 fathers of children with chronic illnesses (response rate: 12.0%, valid response rate: 100.0%), 88 fathers of healthy children (response rate: 16.4%, valid response rate: 97.7%), and a total of 139 participants (response rate: 14.4%, valid response rate: 98.5%). From the valid response count, the analysis included 137 participants (51 fathers of children with chronic illnesses and 86 fathers of healthy children).

### Respondents’ characteristics (Table 1)

The fathers’ mean age was 40.53 ± 5.98 years. The mean age of the first-born child was 7.98 ± 4.90 years. There was a statistically significant difference in the school status of the first-born child, the average age of the first child and annual household income between the fathers of children with chronic illnesses and fathers of healthy children (*p* < 0.001, *p* < 0.001, *p* = 0.012).

### Characteristics of children with chronic illnesses (Table 2)

The most prevalent chronic illnesses of the children were allergic (n = 19, 22.6%) and heart (n = 17, 20.2%) diseases. The most common medical treatment for children was an oral administration of drugs (n = 44, 37.6%) and inhalation (n = 18, 15.4%). Trends in basic attributes and statistical quantities showed that the trends in the present participants are similar to fathers across Japan, indicating that the group is not strongly biased. Since children require relatively advanced treatment at home and routine outpatient visits or hospitalization, it is a group that reflects the characteristics of children with chronic illnesses to a certain degree.

### Influence of the stressor and the father’s mental health (Table 3)

**Table 3:** Relationship between the father’s mental health (K6ab) and the stressor.

Variable	Odds ratio (OR)	95% confidence interval (95% CI)	Significance probability (*p*-valuue)
			n = 137
Children with chronic illness			
Presence	1.000		
Absence	0.286	0.109–0.755	0.011**
Medical treatment required for the children			
< 3 medical treatment	1.000		
≧ 3 medical treatment	4.903	1.368–17.570	0.015**
Number of children			
< 3 children	1.000		
≧ 3 children	2.446	1.011–5.919	0.047*

^a^Mental health(K6): An indicator of mental health, measuring depression.

^b^Dependent variables: K6 high group ( ≧ 5 points): n = 55, K6 low group (5 < points): n = 82.

Model χ2 test (*p* < 0.01).

The Hosmer–Lemeshow test showed a goodness of fit for the logistic regression model (*p* = 0.758).

The discrimination rate between the predicited and actual values was 62.2%.

*p* < .005* *p* < .001**.

Logistic regression analysis results showed that “There are children with chronic illness (OR: 0.286, 95% CI 0.109–0.755, *p* = 0.011)” and “The large number of medical treatments required for the children (OR: 4.903, 95% CI 1.368–17.570, *p* = 0.015)” were significantly associated with poor mental health of the fathers.

### Cognitive structure of fathers’ stress experiences (Table 4)

**Table 4:** Cognitive structure of fathers’ stress experiences of children with chronic illness.

Overall α = .846	Factor		
	I	II	III
			(n = 51)
A sense of well-being and a sense of mission as a father of a chronically diseased child and as a husband α = .937			
As a father, I would do anything for my child’s happiness	.891		
I am happy just to have a child	.879		
For the sake of my children, I will do whatever it takes	.878		
Thanks to my wife, we are able to be a family	.863		
I can do something for my children	.854		
My children are important to me, but as a husband, I want to support my wife	.815		
No matter how hard it is for me, as a husband, I have to think of my wife first	.769		
Because I am a father, I have something to teach my children	.759		
I want to open up the present and the future by myself	.691		
For me, the change in lifestyle associated with the change in children is not bad	.605		
I want to make use of my experience to contribute to society	.408		
A sense of isolation from society and a sense of inability to assess an unpredictable future α = .725			
It’s hard for me not to be able to do my job as I want because of my child		.705	
There’s no one to empathize with me		.621	
I’m sure bad things are waiting for me in the future		.553	
Being a father is a hard role for me		.542	
People around me hurt my feelings unintentionally		.531	
A sense of self-insufficiency as a father of a chronically diseased childα = .761			
I am helpless			.857
I’m giving my child a hard time			.750
In the past, only bad things have happened			.551
Inter-factor correlation			
I	1.000	− .272	.115
II	− .272	1.000	.368
III	.115	.368	1.000

Factor extraction method: Main factor method.

Rotation method: Promax method with Kaiser regularization.

I determined that factor analysis was feasible based on the Kaiser–Meyer–Olkin measure of sampling adequacy, which yielded a value of “0.584,” and Bartlett’s test of sphericity, which resulted in “*p* < 0.000.”

As a result of the factor analysis, the following three factors were extracted as a recognition of the fathers’ stress experiences (overall, α = 0.846): “A sense of self-insufficiency as a father of a chronically diseased child” (three items, α = . 761); “A sense of isolation from society and a sense of inability to assess an unpredictable future” (five items, α = 0.725); and “A sense of well-being and a sense of mission as a father of a chronically diseased child and as a husband” (11 items, α = 0.937).

## Discussion

### Influence of the stressor on the father’s mental health

Logistic regression analysis revealed that being a father of a chronically ill child and the number of medical procedures for the child were significantly associated with stressors on paternal mental health. A previous study^10^ reported that fathers of chronically ill children are more anxious and disappointed than fathers of healthy children and that parenting and daily life are stressors. For the fathers in this study, being the father of a child with a chronic disease was considered to be a stressor for them father, and exposure to stressors in daily life affected their mental health.

### The Cognitive structure of fathers’ stress experiences

The findings revealed that the fathers of children with chronic diseases positively recognize their role as a fathers and their husbands in a positive light despite their negative feelings such as isolation and a sense of self-insufficiency.

In a previous study, the fathers were shocked by the diagnosis of their children’s illness and supported the children and their wives as the fathers or husbands with uncertain anxiety. The fathers had extremely stressful experiences in maintaining family function and building relationships with society^24^. In addition, In a previous study, the results of the analysis of the fathers’ psychological characteristics indicated that they are uncomfortable expressing their emotions, making it challenging to receive adequate support^17^. This suggests that medical practitioners can provide specific assistance measures for the stress experienced by fathers of children with chronic diseases and that nursing care based on a positive psychological approach, focusing on stress cognitive features for a positive recognition of stress experiences, may be useful. For these fathers, based on the positive psychology theory, not only external resources such as information to solve problems and physical support such as money, but also internal resources including emotional support, such as having people listen to them and empathize with them, are important.

### Measures of support based on the positive psychological theory (such as a sense of coherence; SOC) provided to fathers of children with chronic illnesses

The present study showed that when recognizing the stress experienced by fathers of children with chronic illnesses, the support based on the SOC theory may be useful.

Fathers of children with chronic illness incorporate the necessary information to understand the changes in symptoms and possible future incidents. It can also improve the sense of control^38^. It is important to provide psychological support for fathers during the first year of a child’s life following the diagnosis of a chronic illness^25^. Thus, nurses should provide support at an early stage, such as providing accurate information and confirming that the opinions are consistent with those around them, so that fathers can understand the situation.

The fathers of children with chronic illnesses experience negative thoughts; nevertheless, they wish to have positive attitudes and contribute to the society^12^. Fathers are not independent but live in cultural and social settings, being influenced by social factors from family, community, and culture^38^. For SOC, reliable people and the environment are important as they improve the three elements that constitute an SOC. In other words, while fathers are prone to the influence of social interactions, they need to be seen as requiring support, including social support.

The present results showed that the strength of emotional support is significantly correlated with the SOC of the fathers. The psychological characteristics and coping behavior of fathers indicated that they do not complain of distressing symptoms as much as mothers^15^, do not actively express themselves^19^, tend to avoid difficult situations^20^, and do not change their coping behavior unlike mothers who work with others to solve challenging situations^39^. Thus, they might not receive adequate support^24^. Due to the stereotypes of men and fathers in Japan, a father’s ability to manage has been overestimated in the society and by medical personnel^40^. Furthermore, healthcare providers tend to see fathers as “people who support mothers.” As a result, fathers feel that “they were left at the sideline”^41^^,^^42^. Under such a circumstance, based on SOC theory, fathers need internal resources including emotional support, such as having others listen to them and empathize with them^43^, in addition to external resources, such as physical support (information to solve problems and funding).

### Study limitations

Since the present study is a cross-sectional study, it does not indicate a causal relationship. To show the impact of related factors clarified in the present study and other stressors on the mental health of fathers, a longitudinal survey is needed. Since the number of samples was small in the present study, the generalizability of the data is limited. In the future, a different method of recovery for survey responses is necessary, such as including additional sampling requests considering social situations and psychological burden, so that more fathers will agree to participate.

In the present study, we found that a positive psychological approach, focusing on stress cognitive features, for positively recognizing stressful experiences is important for fathers of children with chronic illnesses. In the future, concrete intervention based on a positive psychological theory and verification of the effects are desirable.

## Conclusions

Two points regarding the relationship between mental health and stressors among fathers of children with chronic illnesses and the cognitive structure of stress experiences of fathers who have children with chronic illness were clarified. First, being the father of a child with a chronic disease was considered to be a stressor, and exposure to stressors in daily life affected their mental health. Second, the fathers of children with chronic diseases positively recognize their role as a father and a husband, perceiving it in a positive light despite their negative feelings such as isolation and a sense of self-insufficiency. Therefore, our study data indicated that the measures based on a positive psychology approach could be useful. It also indicated the importance of emotional support based on fathers’ psychological characteristics, allowing fathers to feel empathy and connection with others. Our data will deepen our understanding of this group of fathers and lead to beneficial indications for nursing care.

## Data Availability

The datasets generated and analyzed during the current study are available from the corresponding author on reasonable request.
